# Barriers to Research Utilization among Registered Nurses in Traditional Chinese Medicine Hospitals: A Cross-Sectional Survey in China

**DOI:** 10.1155/2015/475340

**Published:** 2015-11-16

**Authors:** Fen Zhou, Manfred Maier, Yufang Hao, Ling Tang, Hong Guo, Hongxia Liu, Yu Liu

**Affiliations:** ^1^Nursing School, Beijing University of Chinese Medicine, 11 North Third Road, Chaoyang District, Beijing 100029, China; ^2^Department of General Practice, Center for Public Health, Medical University of Vienna, Kinderspitalgasse 15/1, 1090 Vienna, Austria; ^3^Nursing Administration Department, Beijing University of Chinese Medicine Third Affiliated Hospital, 51 Andingmengwai Xiaoguan Street, Chaoyang District, Beijing 100029, China; ^4^Department of Clinical Nursing, Nursing School, Beijing University of Chinese Medicine, 11 North Third Road, Chaoyang District, Beijing 100029, China; ^5^Center for Nursing Research, Nursing School, Beijing University of Chinese Medicine, 11 North Third Road, Chaoyang District, Beijing 100029, China

## Abstract

*Background.* As there might be relevant differences with regard to research utilization in the general hospitals, we aimed to study research utilization among registered nurses working in traditional Chinese medicine hospitals.* Methods.* A total of 648 registered nurses from 4 tertiary-level hospitals in China were recruited for participation. A modified BARRIERS Scale and self-designed questionnaires were used for data collection. Data were analyzed with descriptive statistics, *t*-tests, and one-way ANOVAs and Spearman correlation analysis.* Results.* Overall, items which belong to the subscale “Research” were identified as the most important barriers. Among the individual items, the lack of time on the job was ranked as the top barrier, followed by the lack of knowledgeable colleagues and by overwhelming research publications. Clinical experience, working pressure, job satisfaction, and research experience could be identified as associated factors for barriers to research utilization.* Conclusions.* Registered nurses in traditional Chinese medicine hospitals felt high barriers to research utilization. Reducing registered nurses' working pressure, promoting their positive attitude to nursing, and improving research training might be helpful for increasing research utilization. Close cooperation between clinical and nursing schools or academic research centres might facilitate the necessary change in nursing education and routine.

## 1. Introduction

The term research utilization (RU) has been used since 1969 [1], but without a common definition. Scholars attempted to describe it as an application process of research findings and research methods during daily problem solving [2, 3]. Subsequent to the term evidence-based medicine proposed in 1992 [4], Ingersoll put forward that evidence-based nursing (EBN) is the “conscientious, explicit, and judicious use of theory-derived, research-based information in making decisions about care delivery to individuals or groups of patients reflective of individual needs and preferences” [5]. This definition, therefore, contains three pillars to inform decisions: research (research-based information), patient preference(individual needs and preferences), and clinician expertise (conscientious, explicit, and judicious use). The terms RU and EBN often are used interchangeably although EBN encompasses RU and RU is the undisputed central part and the initial form of EBN [6, 7].

Since 2000, the use of research to inform EBN has been recognized by both the health care community and regulatory agencies as the critical step for improving nursing quality and for provision of safe nursing [8]. This applies also to registered nurses (RNs) who are working in traditional Chinese medicine (TCM) hospitals. However, despite the imperative for RU, many nurses do not apply research findings in their clinical practice. Although nursing has evolved significantly in the past forty years, many gaps between research and clinical practice exist. The International Council of Nurses (ICN) at the occasion of the 100th International Nurses' Day released a statement with the title “Closing the Gap: From Evidence to Action.” The Lancet in response immediately published an editorial pointing out its lateness and implying that contemporary nursing is not evidence based [9, 10]. This is alarming. Therefore, a number of investigations have been conducted to identify the barriers for RU among nurses. In China, for example, the first survey was conducted in Hong Kong already in 2008 [11] and similar articles reported barriers among registered nurses (RNs) in mainland China and Taiwan in 2013 [12–14]. However, their study population was RNs working in general hospitals, and none of them focused on the barriers for RU among RNs who are working in traditional Chinese medicine (TCM) hospitals.

According to the National Health and Family Planning Commission of China, there are six kinds of hospitals in China: general hospitals (15021, 64.83%), TCM hospitals (2889, 12.47%), western medicine and TCM combined hospitals (312, 1.35%), minority national hospitals (208, 0.90%), specialized hospitals (4665, 20.13%), and nursing homes (75, 0.32%) in 2012 [15]. General hospitals usually are dominated by western medicine and nursing, while the TCM hospitals are mainly providing TCM and traditional Chinese nursing (TCN). Different from western nursing, TCN is based on TCM theory and on unique nursing techniques such as acupressure, scrapping, herbal bath, herbal fumigation, and others [16]. Because of the aim for preserving health and of the characteristics to be simple, convenient, cheap, and effective, TCM and TCN in China are very popular. As of 2010, there were 1.86 million RNs working in TCM hospitals [17].

Compared with general hospitals in China, the ratio of doctors (including both western medicine and TCM) to nurses of TCM hospitals is low, only 1 : 0.98 (in general hospitals it is 1 : 1.28), and the ratio of patients to nurses is 1 : 0.39 (in general hospitals it is 1 : 0.47) [18]. Given the same level of professional autonomy, these differences imply that nurses of TCM hospitals might be busier. In addition, Jue's investigation showed that only 23.6% of nurses with a bachelor or higher education background work in TCM hospitals, and most of them obtained their degree from non-full-time education, for example, distance learning [19]. Hence, there might be some differences of relevance with regard to barriers for RU between RNs from the two kinds of hospitals. Therefore, this study aimed to explore the barriers for RU among RNs in the TCM hospital.

## 2. Methods

### 2.1. Study Design and Sample

This cross-sectional study used a convenience sample of RNs employed in four TCM hospitals in Beijing, China. RNs were defined as officially certified for nursing practice and as being engaged in clinical nursing practice regardless of their educational degree. Nurses from all shifts (including those on night duty) were approached. The inclusion criteria were undergraduate education regardless of through what form of education (including college undergraduate education and in-service continuous nursing education) and all qualified RNs were included. Student nurses were excluded. The hospitals investigated are tertiary-level hospitals: three of them are affiliated with a medical university; one is attached with another academic institution.

The study was conducted with the approval from the institutional review board of Beijing University of Chinese Medicine (approval number 2015BZHYLL0407).

### 2.2. Data Collection

The tools used in this survey included a self-designed questionnaire and a modified BARRIERS Scale [20]. The self-designed questionnaire contained demographics (age, gender), first education level, duration of clinical experience in health care, position, and working department. In addition, two questions with options were used to evaluate the self-perceived working pressure and attitude toward nursing: (1) How do you like nursing (like, neither like nor dislike, or dislike)? (2) How do you think about the pressure of your nursing work (no pressure, a little, just so-so, moderate, or strong)? Research experience was assessed by being involved in scientific research programs (including design, practice, and write) or not with two options: yes (means participate with one step of them) and no (means did not participate with all of them).

The BARRIERS Scale was developed by Funk et al. in 1991 as a measurement tool with which to identify barriers to research utilization in practice [20]; since then, it has been applied in 63 surveys on nursing from 14 countries [21]. In 2006, Thompson et al. translated it into Chinese and reported that the Chinese version's content validity value was 0.98, and those of the subscales were 0.71 to 0.88 [22]. We made a little modification to the BARRIERS Scale (Chinese version): the item “the nurse is unwilling to try/change new ideas” in the original scale was split into two items: “the nurse is unwilling to change practice” and “the nurse is unwilling to try new ideas” to improve clarity. This modified BARRIERS Scale is therefore composed of 30 items (original 29) with four subscales: Nurse Characteristic (“Nurse,” 9 items), Quality of Research (“Research,” 7 items), Organization Characteristics (“Setting,” 8 items), and Presentation and Accessibility of Research (“Presentation,” 6 items). Each item provides five-point Likert-type choices, from 1, “to no extent,” to 5, “to a great extent.” In our pilot twenty-sample survey, Cronbach's alpha values were 0.88, and those of the subscales were 0.70 to 0.79.

Each hospital had staff from our research group in charge of distribution and collection of the questionnaires. Filling the questionnaires was anonymous. Each participant received a small gift (soap) as reward in order to improve the response rate. The questionnaires were immediately sent back to our office. After three months' recruitment, a total of 720 RNs were invited to participate.

### 2.3. Data Analysis

RNs were classified into three subgroups depending on their different level of clinical experience (<10 years, 10~15 years, and >15 years) and first educational level (diploma, associate, bachelor, and higher degree) and two subgroups depending on nursing administrator or not and research experience or not, respectively. Self-perceived working pressure and attitude to nursing (job satisfaction) were considered ordinal variables. Scores of 4 (to moderate extent) and 5 (to great extent) for each item of the BARRIERS-tool were combined, which indicates that respondents perceived moderate or great barrier with this item. And we applied mean scores to report each subscale.

SPSS software version 18.0 (SPSS Inc., Chicago, IL, USA) was used for data analyses. Frequency, percentage, median, and range were applied in the description of variables. The relationships between self-perceived working pressure and attitude to nursing related to the BARRIERS-tool were described by Spearman's rho (*ρ*); the differences between groups of RNs were determined by *t*-tests and one-way ANOVAs (LSD methods used for pairwise comparison). The level of statistical significance was set at *P* < 0.05.

## 3. Results

### 3.1. Characteristics of the Participants

Out of 720 RNs with undergraduate education employed at the four hospitals, a total of 680 questionnaires were returned (response rate 95.29%). Thirty questionnaires had to be excluded because of incomplete data, resulting in a final sample size for analysis of 648.

The demographic characteristics, self-perceived working pressure, attitude to nursing/job satisfaction, and research experience of respondents are shown in Table 1. Their mean age was 30.54 years and their median clinical experience was 7 years. Although all the participants held the highest qualification as an undergraduate, almost half of them obtained it later from their original lower education (diploma) (42.6%, *n* = 276). In addition, 3.55% (*n* = 23) were studying toward master degree, and 10.3% (*n* = 67) respondents worked in an administrative position.

As can be seen from Table 1, 70.8% (*n* = 459) of respondents perceived their working pressure as moderate and strong and 28.4% (*n* = 184) disliked their nursing work. Almost half of the participants (41.5%; *n* = 269) had no research experience.

### 3.2. Perceptions of Barriers to Research Utilization

The results of the BARRIERS Scale and its 4 subscales are shown in Table 2. The scores ranged from 2 to 5, with a mean of 3.08 for the overall score (SD = 0.48; 95% CI 3.04 to 3.12). The subscale of “Research” was the highest one (mean = 3.27, SD = 0.57), followed by the “Presentation” (mean = 3.08, SD = 0.6), the third one is “Setting” (mean = 3.08, SD = 0.58), and the lowest one is “Nurse” (mean = 2.93, SD = 0.53). More than half of the respondents scored 7 items as 4 or 5, and these 7 items were dispersed on four subscales.  Table 3 shows the results for the 30 individual items of the BARRIERS Scales and their rank order. The top one was pertaining to the “Setting,” while the bottom two items were all pertaining to the “Nurse.”

### 3.3. Associations

Although no Spearman's correlation above *ρ* = 0.4 was found, two factors still showed statistical significance (Table 4):

(1) Working pressure was correlated with the total scale (Spearman's *ρ* = 0.23, *P* < 0.001) and with all the subscales (range of Spearman's *ρ* was from 0.15 to 0.20, *P* < 0.001), indicating that the higher the pressure was perceived, the greater the barriers were felt.
(2) Attitude to nursing/job satisfaction was found to have a positive correlation with the total scale (Spearman's *ρ* = 0.11, *P* < 0.05), with “Nurse” (Spearman's *ρ* = 0.11, *P* < 0.05), and with “Setting” subscales (Spearman's *ρ* = 0.13, *P* < 0.05), showing that the worse the attitude is, the greater the barriers were felt.

### 3.4. Comparisons of Groups of RNs

Significant statistical results were found for different subgroups of RNs (Table 5):

(1) Different clinical experience resulted in statistically significant differences in scores of both the total scale (*F* = 7.95, *P* < 0.001) and subscales (the range of *F* from 3.50 to 11.90, *P* < 0.05). After pairwise comparison, a clinical experience of <10 years had higher scores compared with those of 10~15 years and with >15 years in both the total scale and two subscales (Presentation and Research). RNs with a clinical experience of <10 years achieved higher scores than those with an experience of >15 years in “Nurse” and “Setting” subscales. The shorter the clinical experience, the higher the barriers' scores. The results indicate that clinical experience is a significant factor influencing perceptions of barriers.
(2) Compared with RNs having research experience, those without research experience perceived higher scores not only in the total scale (*t* = 3.09, *P* < 0.05) but also in three subscales (Nurse, Setting, and Presentation; the range of *t* from 2.59 to 4.06, *P* < 0.05). This indicates that nurses with research experience felt lower barriers than those without.

## 4. Discussion

This survey among RNs from four (first-class hospitals as evaluated by the Minister of Health) TCM hospitals for the first time identified existing barriers to RU and their associated characteristics.

### 4.1. High Working Pressure and Low Research Experience as the Main Characteristics

It is worth noting that more than 70 percent of RNs perceived moderate and great working pressure (Table 1). The reason might be the low nurse-to-patient ratio in China [23]. Additionally, despite their undergraduate education, still almost half of the participants in this survey had no experience with research (Table 1). However, all undergraduates in nursing are supposed to have some research exposure/experience. These findings demonstrate that RNs working in TCM hospital suffer from working pressure and that undergraduate education needs to improve the research training part.

### 4.2. Perceived Barriers Are Many but Research-Pertaining Barriers Were the Highest

The scores for the total and for the subscales of BARRIERS showed significant and various barriers for RNs working in TCM hospitals (Table 2). Barriers were at approximately the same level as those in the general hospitals in mainland China [14], but higher than in western countries [24–26] and in Hong Kong [11]. Compared with other countries, RU seems rather new for RNs from TCM hospitals, which implies RU is still at the preliminary stage in TCN field. In addition, implementation of RU should be based not only on some research knowledge and skills, but also on some external factors, for example, on an official support for RU by the employer or for an EBN culture and context [27]. Therefore, RNs from TCN might feel higher barriers from all sides. Among the subscales, it was surprising that the highest perceived barriers were related to the research subscale (Table 2) but not related to “Setting” shown in previous studies [12–14, 24]. Possible explanations for this finding may be that much of the research literature in Chinese is of poor quality in terms of methodology or reporting [28, 29]. On the other hand, it is not easy for RNs to get access to the high quality researches from western countries due to the language barrier. Further, none of the hospitals provides access to databases with evidence-based summaries. Participants can only get access to the school of nursing, if they want. Hence, this also enhances the barrier for RU in their daily clinical life. Overall, the low quality of research appears to be the top item of barriers for RU in RNs in TCN. In other words, when promoting the development of RU in the field of TCN research of high quality would be helpful.

### 4.3. Insufficient Time to Implementation, Isolation from Knowledgeable Colleagues, and Overwhelming Research Information as the Top Three Greatest Barriers

Lack of time on the job was a major barrier, followed by isolation from knowledgeable colleagues and by overwhelming research information (Table 3). 70.8% of RNs perceived moderate or strong working pressure (Table 1). Implementing research findings may require the practitioner to change their working routine, which is a challenge for RNs [30]. Furthermore, daily busy working schedules apparently may make it impossible to regularly read increasing numbers of publications. Hence, self-perceived working pressure was found to have a positive relationship with both total and all subscales of BARRIERS (Table 4).

### 4.4. Factors Related to the Perceptions of Barriers

Clinical experience was found to be an associated factor with the perceptions of barriers. RNs with less than 10 years of clinical experience perceived the greatest barriers followed by those with 10~15 years; the smallest were those with more than 15 years (Table 5). Therefore, advanced clinical experience may help nurses to identify the needs for improvement or to evaluate the applicability of new research evidence.

Further, the job satisfaction apparently influences the perceived total, “Nurse,” and “Setting” barriers: the higher the satisfaction is, the less the barriers are felt. There were 28.4% of RNs in this survey that were dissatisfied with their work (Table 4). This seems to be a high percentage which might also influence professional performance [31, 32].

RNs with research experience have lower scores in the total and all subscales of BARRIERS (Table 5). Similar findings were also reported in Chen et al.'s [12] and Wang et al.'s studies [14]. In addition, the result of a similar Swedish survey, where RNs that accepted nursing program without research methodology perceived higher barriers, also supported this finding [24]. Positive attitude to RU, necessary skills and knowledge on RU, and research management skills also could be developed when conducting or participating in research [33].

### 4.5. Limitations

This survey used a convenience sample focusing on academic TCM hospitals in Beijing, China. Therefore, our results may not be representative for all RNs in TCM hospitals. Only sociodemographic data and a few potential associations were collected for analysis. Obviously, there might be other potential factors affecting RU. Hence, randomized sampling in various TCM hospitals considering additional factors is needed.

### 4.6. Strengths of Our Study

To the best of our knowledge, this survey was the first one conducted focusing on RNs with undergraduate education in TCM hospitals. Our study enjoyed a very high response rate (95.29%), which might be attributed to the small gift and the strong support of nurse managers from the four hospitals included. Further, we used a standardized and validated instrument to assess the main questions of this survey: the barriers to RU.

### 4.7. Implications of Our Results

We think that RU should not only be seen as an individual professional responsibility; for example, it needs a group of motivated professionals with knowledgeable peers [34]. Further, nursing schools should provide more research training and should support research experiences of high quality for nurse students and nurses who are involved in in-service education [35]. Hospitals or medical institutions should reconsider optimizing the nurse-to-patient ratio in order to reduce the burden of routine work and to improve job satisfaction. Finally, close cooperation between clinical and nursing schools or academic research centres might facilitate or even enable the proposed change in nursing education and routine. For example, front-line nurses could propose clinical questions or problems which need to be solved or improved; advanced nurses, nursing researchers, or academic personnel could plan and conduct the research exercise and could provide useful answers. During implementation of the research results, this multilevel team could cooperate and complement each other and could apply some models such as the Stetler model, the Iowa model, or the John Hopkins Evidence-Based Practice Model [36]. Designing a study to explore the effect of a team including clinical and academic nursing personnel on RU might be a thought for future research. A mixed research design, combining quantitative and qualitative evaluation methods, could be more comprehensive in order to appraise the results.

## 5. Conclusions

This survey with 648 RNs from four TCM hospitals identified various barriers to RU. The research-pertaining barriers were the leading ones, in particular insufficient time, lack of knowledgeable colleagues, and overwhelming numbers of research publications to be read. Compared to the RNs in western countries, our participants perceived higher barriers to RU. Low job satisfaction and lack of research training are associated with perceptions of barriers.

## Figures and Tables

**Table 1:** The participants' demographic characteristic, self-perceived working pressure, and attitude to nursing (*N* = 648).

Variable	*N* (%)
Age (years)	Mean (SD): 30.54 (6.99)
Clinical experience (years)	Median (range): 7 (1~37)
<10 years	432 (66.67)
10~15 years	113 (17.44)
>15 years	103 (15.89)
First education degree	
Diploma	276 (42.59)
Associate	225 (34.72)
Bachelor	147 (22.69)
In-service master degree	23 (3.55)
Working department	
Medical/gerontological	220 (33.95)
Surgical/operating theatre	217 (33.49)
ICU/critical care	77 (11.88)
Obstetric/gynaecologic	25 (3.86)
Pediatric	27 (4.17)
Others (e.g., A&E and day care centre)	82 (12.65)
Nursing administrator	
Yes	67 (10.34)
No	581 (89.66)
Self-perceived working pressure	
No pressure	1 (0.15)
A little	1 (0.15)
Just so-so	187 (28.86)
Moderate	323 (49.85)
Strong pressure	136 (20.99)
Attitude to nursing	
Like	75 (11.57)
Neither like nor dislike	389 (60.03)
Dislike	184 (28.40)
Research experience	
Yes	379 (58.49)
No	269 (41.51)

**Table 2:** Means and Standard Deviations of the BARRIERS Scale and its subscales (*N* = 648).

Scale or subscales	Mean (SD)	95% CI
BARRIERS Total Scale	3.08 (0.48)	3.04~3.12
Nurse	2.93 (0.53)	2.89~2.97
Setting	3.08 (0.58)	3.03~3.12
Presentation	3.08 (0.60)	3.04~3.13
Research	3.27 (0.57)	3.23~3.32

**Table 3:** Barriers items in rank order (*N* = 648).

Items	Subscale	Rating item as		Rank order
		moderate or great		
		barrier *N* (%)		
There is insufficient time on the job to implement new ideas	Setting	381	58.8	1
The nurse is isolated from knowledgeable colleagues with whom to discuss the research	Nurse	372	57.5	2
The amount of research information is overwhelming	Research	348	53.7	3
The nurse does not have time to read research	Setting	345	53.2	4
The research is not relevant to the nurse's practice	Research	343	52.9	5
The research has methodological inadequacies	Research	341	52.6	6
The facilities are inadequate for implementation	Setting	338	52.1	7
The nurse is uncertain whether to believe the results of the research	Research	301	46.4	8
The nurse does not feel capable of evaluating the research	Nurse	288	44.4	9
Research reports/articles are not published fast enough	Research	287	44.3	10
The nurse does not feel she/he has enough authority to change patient care procedures	Setting	280	43.2	11
Research reports/articles are not readily available	Presentation	271	41.8	12
The literature reports conflicting results	Research	271	41.8	12^*∗*^
The relevant literature is not compiled in one place	Presentation	269	41.5	14
There is not a documented need to change practice	Nurse	254	39.2	15
The statistical analyses are not understandable	Presentation	234	36.1	16
Implications for practice are not made clear	Presentation	224	34.5	17
The conclusions drawn from the research are not justified	Research	222	34.3	18
The nurse does not see the value of research for practice	Nurse	217	33.5	19
Physicians will not cooperate with implementation	Setting	198	30.8	20
The research is not reported clearly and readably	Presentation	193	29.7	21
The nurses feel the results are not generalisable to their own setting	Setting	184	28.4	22
Other staffs are not supportive of implementation	Setting	139	21.4	23
The nurse sees little benefit for himself or herself	Nurse	130	20.1	24
The research has not been replicated	Presentation	125	19.3	25
The nurse feels the benefits of changing practice will be minimal	Nurse	114	17.5	26
Administration will not allow implementation	Setting	113	17.4	27
The nurse is unaware of the research	Nurse	110	17	28
The nurse is unwilling to change new ideas	Nurse	62	9.6	29
The nurse is unwilling to try new ideas	Nurse	62	9.6	30

^*∗*^Two items had the same percent ranking.

**Table 4:** Factors influencing perceptions of barriers from univariate analysis: ordinal variables (*N* = 648).

	Self-perceived working pressure	Attitude to nursing
BARRIERS Total Scale	*ρ* = 0.234^*∗*^	*ρ* = 0.106^*∗*^
Nurse	*ρ* = 0.195^*∗*^	*ρ* = 0.106^*∗*^
Setting	*ρ* = 0.145^*∗*^	*ρ* = 0.130^*∗*^
Presentation	*ρ* = 0.169^*∗*^	*ρ* = 0.052
Research	*ρ* = 0.145^*∗*^	*ρ* = 0.044

Note: Spearman correlation statistics were used due to ordinal variables.

^*∗*^ *P* < 0.05.

**Table 5:** Factors influencing perceptions of barriers from univariate analysis: continuous variables (*N* = 648).

	Clinical experience (years)			Research experience	
	1	2	3	Yes	No
	Mean (SD)			Mean (SD)	
BARRIERS Total Scale	3.13 (0.46)	3.02 (0.45)	3.08 (0.48)	3.01 (0.45)	3.13 (0.49)
	*F* = 7.951^*∗*^			*t* = 3.088^*∗*^	
	LSD pairwise comparison: 1 : 2^*∗*^; 1 : 3^*∗*^				

Nurse	2.97 (0.52)	2.89 (0.48)	2.83 (0.57)	2.87 (0.48)	2.98 (0.55)
	*F* = 3.504^*∗*^			*t* = 2.586^*∗*^	
	LSD pairwise comparison: 1 : 3^*∗*^				

Setting	3.12 (0.57)	3.01 (0.55)	2.97 (0.63)	2.97 (0.54)	3.15 (0.59)
	*F* = 3.815^*∗*^			*t* = 4.059^*∗*^	
	LSD pairwise comparison: 1 : 3^*∗*^				

Presentation	3.15 (0.58)	3.02 (0.58)	3.08 (0.66)	3.14 (0.61)	3.00 (0.58)
	*F* = 11.895^*∗*^			*t* = 2.978^*∗*^	
	LSD pairwise comparison: 1 : 2^*∗*^; 2 : 3^*∗*^; 1 : 3^*∗*^				

Research	3.33 (0.55)	3.19 (0.56)	3.16 (0.66)	3.027 (0.57)	3.28 (0.58)
	*F* = 5.220^*∗*^			*t* = 0.151	
	LSD pairwise comparison: 1 : 2^*∗*^; 1 : 3^*∗*^				

*Note.* 1: clinical experience less than 10 years; 2: clinical experience from 10 to 15 years; 3: clinical experience more than 15 years; ^*∗*^ *P* < 0.05.
